# Network meta-analysis-highly attractive but more methodological research is needed

**DOI:** 10.1186/1741-7015-9-79

**Published:** 2011-06-27

**Authors:** Tianjing Li, Milo A Puhan, Swaroop S Vedula, Sonal Singh, Kay Dickersin

**Affiliations:** 1 Johns Hopkins Bloomberg School of Public Health, 615 N. Wolfe Street, Mail Room W5010, Baltimore, Maryland, 21212, USA; 2 Johns Hopkins Medical Institutions, 1830 E. Monument St, Suite 8063, Baltimore, Maryland, 21212, USA

## Abstract

Network meta-analysis, in the context of a systematic review, is a meta-analysis in which multiple treatments (that is, three or more) are being compared using both direct comparisons of interventions within randomized controlled trials and indirect comparisons across trials based on a common comparator. To ensure validity of findings from network meta-analyses, the systematic review must be designed rigorously and conducted carefully. Aspects of designing and conducting a systematic review for network meta-analysis include defining the review question, specifying eligibility criteria, searching for and selecting studies, assessing risk of bias and quality of evidence, conducting a network meta-analysis, interpreting and reporting findings. This commentary summarizes the methodologic challenges and research opportunities for network meta-analysis relevant to each aspect of the systematic review process based on discussions at a network meta-analysis methodology meeting we hosted in May 2010 at the Johns Hopkins Bloomberg School of Public Health. Since this commentary reflects the discussion at that meeting, it is not intended to provide an overview of the field.

## Introduction

Systematic reviews use explicit, pre-specified methods to identify, appraise, and synthesize all available evidence related to a clinical question. When appropriate, systematic reviews may include a meta-analysis, that is, the statistical combination of results from two or more separate studies. Some systematic reviews compare only two interventions, in which a conventional pair-wise meta-analysis may be conducted, while others examine the comparative effectiveness of many or all available interventions for a given condition. When the comparative effectiveness of a range of interventions is of interest, appropriate statistical methodology must be used for analysis.

**Figure 1:**
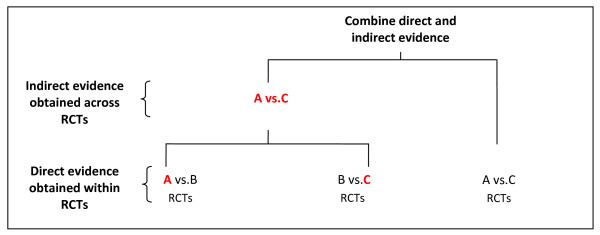
**Illustration of a network meta-analysis that combines direct evidence obtained within RCTs (A vs. B, B vs. C and A vs. C), and indirect evidence obtained across RCTs through a common comparator (A vs. B and B vs. C)**.

Also called mixed treatments comparison or multiple treatments comparison meta-analysis, network meta-analysis expands the scope of a conventional pair-wise meta-analysis by analyzing simultaneously both direct comparisons of interventions within randomized controlled trials (RCTs) and indirect comparisons across trials based on a common comparator (e.g., placebo or some standard treatment) [1-5]. In the simplest case, one may be interested in comparing two interventions A and C. Indirect evidence can be obtained from RCTs of either A or C versus a common comparator B (Figure 1), keeping intact the randomized comparisons within the RCTs [1-5]. When both direct and indirect evidence are available, the two sources of information can be combined as a weighted average when appropriate. Data structure of this type can be extended to k-comparisons to facilitate simultaneous inference regarding all available treatments, and to provide evidence for selecting the best of several treatment options. Many assumptions behind network meta-analysis methods appear to be similar to those made in standard pair-wise meta-analysis [6]. But as for a conventional pair-wise meta-analysis, the methodology for network meta-analysis must be carefully developed and rigorously evaluated before the technique is applied widely.

Despite a recent flurry of publications related to network meta-analyses [7], only a handful of articles have focused on key methodological issues and most of these have covered statistical approaches [2-4,8-16]. In May 2010, we hosted a meeting on network meta-analysis methodology at the Johns Hopkins Bloomberg School of Public Health. Vibrant discussions over the course of the meeting led us to identify major methodological questions concerning network meta-analysis and to propose a research agenda for the future. This article reflects discussion at the meeting and is not intended to provide an overview of the entire field.

## Discussion

Using statistical methods to combine findings from individual studies in a systematic review can provide useful information for clinical decision-making. To minimize error and ensure validity of findings from meta-analyses, the systematic review, whether it involves a standard, pair-wise meta-analysis or a network meta-analysis, must be designed rigorously and conducted carefully. Aspects of designing and conducting the systematic review include defining the review question, specifying eligibility criteria, searching for and selecting studies, assessing risk of bias and quality of evidence, conducting a meta-analysis, and interpreting and reporting findings [6]. The following sections discuss methodologic challenges and research opportunities for network meta-analysis relevant to each aspect of the systematic review process.

### Define the review question and eligibility criteria

A well-formulated, clearly defined, answerable research question guides the eligibility criteria and the overall research protocol. Eligibility criteria combine aspects of the clinical question (e.g., Population, Interventions, Comparisons, and Outcomes) and specifications of the types of studies that have addressed this question [6]. Although the questions asked in pair-wise meta-analysis and network meta-analysis on a topic are different, the same interventions and comparisons may be examined, and these may be defined broadly or narrowly in both. For example, in both cases, one would want to define whether both drugs and behavioral interventions would be included, and if so, which ones. One would also want to define whether a different dose or regimen of the same treatment should be considered as the same or separate interventions.

Different specification of eligibility criteria may result in differences in the structure or extent of a network, leading to discrepant findings for network meta-analyses on the same topic. This is because different combinations of direct and indirect evidence, some independent and some overlapping, contribute to the comparisons and estimates of treatment effect [3,5]. Certain interventions, for example, interventions that are no longer in use, or placebos, may not be of primary interest but may be included in the network meta-analysis if they provide information concerning the interventions of interest through indirect comparisons. In a recent example, discordant conclusions were drawn from two systematic reviews that utilized direct and indirect evidence regarding the comparative effectiveness of second generation anti-depressants for major depression disorder [17,18]. One reason for the discrepancy was the difference in how the networks were defined [17-19]. One systematic review did not include placebo-controlled trials [17]. It is currently not possible to make general statements on the impact that different eligibility criteria may have on the validity of findings from a network meta-analysis.

Eligibility criteria in a review of harms may be different from a review of effectiveness because there might be limited data related to harm or adverse effects in a trial [20]. Methodologic research is needed to establish the role of non-randomized studies within a network meta-analysis evaluating harms associated with interventions.

### Search for and select studies

To ensure that all relevant studies are identified, the network meta-analyst could search *de novo *for all relevant studies, but this would waste valuable resources if good systematic reviews with comprehensive searches already exist. To conserve valuable resources, one might consider using data identified through existing high quality systematic reviews of relevant pair-wise treatment comparisons provided the searches in the existing reviews are up-to-date. Empirical research is needed on the trade-offs associated with the two approaches to identify trials for a network meta-analysis. Such work will provide guidance for the network meta-analyst in choosing between conducting a new, comprehensive search or using existing searches.

As it is the case with a conventional pair-wise meta-analysis, the validity of findings from a network meta-analysis depends upon whether all eligible trials were identified and included in the analysis. Regardless of whether one conducts *de novo *searches or depends on existing systematic reviews, including a non-random or selective subset of all eligible trials in the analysis may introduce selection bias in the treatment effect estimates. Various forms of reporting biases have been identified in the literature [21]. As a consequence of reporting biases, for example, the network meta-analyst may fail to identify certain trials or when trials are identified, fail to retrieve data on outcomes relevant for analysis. One way that certain reporting biases are addressed is by conducting a search of multiple data sources for trial data. The various data sources that may be searched to retrieve trial data include published data, conference abstracts and other sources of grey literature, clinical trial registers, internal company reports, reviews of trials by regulatory agencies, and requesting trial investigators for individual patient data. Network meta-analysis involving both drug and non-drug interventions, for example, may be affected disproportionately if industry-sponsored trials are subject to greater reporting biases than other studies. Similarly, the internal validity of network meta-analysis of drug interventions may be affected if placebo-controlled trials are subject to greater reporting biases than active-controlled trials [22]. Methodological research is needed to examine the impact of various reporting biases and the use of multiple sources of trial data on the design, analysis, and findings from network meta-analyses.

### Assess risk of bias and quality of evidence

The assessment of the risk of bias and its consideration in the network meta-analysis is far more challenging than in conventional meta-analysis. Risk of bias refers to the problems with the design and execution of individual trials that raise questions about the validity of their findings [6]. A fundamental difference between a conventional pair-wise meta-analysis and network meta-analysis is that a conventional pair-wise meta-analysis yields only one pooled effect estimate whereas a network meta-analysis yields more than one pooled effect estimate. Thus, while bias in the effect estimate from any single trial affects a single pooled effect estimate in a conventional meta-analysis, it may affect several pooled effect estimates obtained in a network meta-analysis. For example (Figure 1), the risk of bias for trials contributing to the direct comparison within a network may be low (e.g., all A vs. C trials described adequate masking), but the risk of bias for trials contributing to the indirect comparison may be high (e.g., some A vs. B or B vs. C trials reported no masking). In addition, the risk of bias may differ across different regions within the network of interventions being examined. Future methodological research should address ways to deal with such variation in risk of bias between direct and indirect comparisons and across the network. Specifically, such research may examine the impact of risk of bias in an individual trial on the network meta-analytic effect estimates, identify the biases specific to the network meta-analysis context that need to be considered, develop methods to assess, summarize and present the variation in risk of bias across the network, and use empirical research to postulate guidance for network meta-analysts on incorporating bias assessments in statistical analyses. Finally, methodological research may also examine whether network meta-analysis offers a potential method for identifying and adjusting for biases within included trials [10,15,23].

### Conduct quantitative evidence synthesis

Several statistical methods are being used to implement network meta-analysis, for example, the adjusted indirect comparison method with aggregate data, meta-regression, hierarchical models, and Bayesian methods [1]. Some approaches provide better flexibility than others in adjusting for covariates and in ranking multiple interventions. Most network meta-analyses in the current literature use a single approach and the comparative performance of different approaches has not been studied in detail. Future methodological studies may evaluate the utility and robustness of various statistical methods, and identify circumstances in which specific methods or models are more efficient and appropriate than others.

Factors such as the total number of trials in a network, number of trials with more than two comparison arms, *heterogeneity *(i.e., clinical, methodological, and statistical variability within direct and indirect comparisons), *inconsistency *(i.e., discrepancy between direct and indirect comparisons), and bias may influence effect estimates obtained from network meta-analyses. Heterogeneity, inconsistency, and bias may propagate through a network of trials, and may affect the estimates differentially across regions of the network. A range of methods to detect, quantify and deal with heterogeneity, inconsistency, and bias has been proposed [10-12,15,23]. Evaluating the performance of the different methods, through simulations and empirical studies, is critical before they become widely available.

Most network meta-analyses to date use WinBUGs software, which is limited in functionality and accessibility to the non-statistician. New software is needed that balances user-friendliness with statistical sophistication and provide built-in methodological guidance. In addition, new software should be able to handle in a coherent manner different types of outcomes (e.g., continuous outcomes, binary outcomes), multiple outcomes, outcomes at different follow-up times and simultaneously carry out pair-wise and network meta-analysis.

With availability of the new, easy to use software, concerns arise about network meta-analysis being undertaken and implemented inappropriately. Thus, systematic reviewers should be educated to identify potential research questions where network meta-analysis may be appropriate, and where it is not, including the situation where the evidence is sparse.

### Interpret results and report findings

Presenting and communicating complex findings from a network meta-analysis in an accessible and understandable format is challenging. It is critical to report all pair-wise effect estimates together with the associated confidence or credible intervals, depending on the statistical model used (i.e., frequentist or Bayesian model). Probability statements could be made about the effectiveness of each treatment [24]. For example, for each treatment, one can calculate the probability that the treatment is the best, second best, or third best among all treatments. Such probability statements should be interpreted carefully since the difference between treatments might be small and not clinically meaningful.

In addition to the estimates of treatment effects, uncertainty, clinical and methodological characteristics, and potential biases within included trials must be conveyed. A careful assessment of the body of evidence and a thoughtful discussion of the potential impact of trial-specific biases on the effect estimates in a network meta-analysis can maximize transparency and avoid errors in interpretation. Using the hypothetical example described in a preceding section, if the preponderance of evidence within the network is constituted by trials that did not report masking, interpreting effect estimates from a network meta-analysis of such trials should be tempered by a discussion on the impact of potential bias due to inadequate masking. Values and preferences from potential evidence users should be considered in interpretation. Guidelines may be developed, based on methodological research, to establish standards for reporting network meta-analyses. Although a recent survey identified nearly 100 published network meta-analyses published between 2000 and 2007 [7], many peer reviewers are relatively uneducated in these methods. Guidance may be developed to aid rigorous peer review of findings from network meta-analyses submitted to medical journals.

## Conclusions

This commentary summarizes the methodologic challenges and areas of research for network meta-analysis relevant to each aspect of the systematic review process based on discussions at a meeting. It is not intended to provide a comprehensive overview of the field. Network meta-analysis holds promise to provide evidence on comparative effectiveness that is valuable for clinical decision-making because it allows comparisons of interventions that may not have been directly compared in head-to-head trials. Collaborative efforts between epidemiologists, statisticians, clinicians and others are necessary for developing, implementing and evaluating methods for network meta-analysis. The extent to which the medical community accepts network meta-analysis will depend on how convincingly methodological research demonstrates the validity of the evidence and its ease of interpretation for decision-makers.

## Competing interests

TL, MP, and KD reported receiving salary support though a grant to the Johns Hopkins Bloomberg School of Public Health from the National Eye Institute, National Institutes of Health to conduct the study and to write the manuscript. The grant also supports TL, MP, and KD to travel to meetings related to the study. SV and SS declared that they have no financial or non-financial competing interests.

## Authors' contributions

TL has full access to all of the transcriptions of meeting discussions, and takes responsibility for the integrity of the manuscript. TL, MP, SV, SS, and KD participated in the study conception, design, and analysis, and interpretation of findings. TL, MP, and SV drafted the manuscript. SS and KD reviewed and edited the manuscript for important intellectual content. KD obtained funding for this study, and supervised the work. TL and KD also provided administrative and material support. Meeting contributors either presented their current research and ideas, or provided insightful comments during the meeting, and sent constructive feedback to the manuscript draft. All authors read and approved the final manuscript.

## Pre-publication history

The pre-publication history for this paper can be accessed here:

http://www.biomedcentral.com/1741-7015/9/79/prepub
